# Network meta-analysis of first-line thrombectomy strategy for acute posterior circulation strokes: a preliminary evaluation for combined approach

**DOI:** 10.3389/fneur.2023.1279233

**Published:** 2023-11-03

**Authors:** Gengfan Ye, Ruoyao Cao, Pandi Chen, Hongcai Wang, Dongfeng Wang, Maosong Chen, Zhenqiang Li

**Affiliations:** ^1^Department of Neurosurgery, Ningbo Medical Center Lihuili Hospital, Ningbo, China; ^2^Department of Radiology, Beijing Hospital, National Center of Gerontology; Institute of Geriatric Medicine, Chinese Academy of Medical Sciences, Beijing, China; ^3^Graduate School of Peking Union Medical College, Beijing, China

**Keywords:** stroke, posterior circulation, frontline strategy, stent retriever, aspiration, combined technique

## Abstract

**Objective:**

Thrombectomy may provide superior results compared to best medical care for acute posterior circulation strokes (PCS). Contact aspiration (CA), stent retriever (SR), and combined SR + CA (SRA) are commonly employed as first-line techniques. However, the optimal strategy and the role of SRA remain uncertain.

**Methods:**

Systematic searching was conducted in three databases (PubMed, Embase, and Cochrane). Network meta-analyzes were performed using random-effects models. The reperfusion and clinical outcomes were compared. Pooled outcomes were presented as odds ratios (OR) with 95% confidence intervals (CI). Rankograms with surface under the cumulative ranking curve (SUCRA) were calculated.

**Results:**

Seventeen studies were included, involving a total of 645 patients who received first-line CA, 850 patients who received SR, and 166 patients who received SRA. Regarding final recanalization outcomes, both first-line SRA (OR = 3.2, 95%CI 1.4–11.0) and CA (OR = 2.1, 95%CI 1.3–3.7) demonstrated superiority over SR in achieving successful reperfusion [modified Thrombolysis In Cerebral Infarction (mTICI) 2b-3], with values of SUCRA 91.1, 58.5, and 0.4%, respectively. In addition, first-line SRA showed an advantage in achieving final mTICI 2c/3 compared to CA (OR = 3.6, 95%CI 0.99–16.0) and SR (OR = 6.4, 95%CI 1.3–35.0), with SUCRA value of 98.0, 44.7, and 7.2%, respectively. Regarding reperfusion outcome after the first pass, SRA also achieved a higher rate of mTICI 3 than SR (OR = 4.1, 95%CI 1.3–14.0), while CA did not (SUCRA 97.4, 4.6, 48.0%). In terms of safety outcomes, first-line CA was associated with a lower incidence of symptomatic intracranial hemorrhage (sICH) compared to SR (OR = 0.38, 95%CI 0.1–1.0), whereas the SRA technique did not (SUCRA 15.6, 78.6, 55.9%). Regarding clinical prognosis, first-line CA achieved a higher proportion of functional independence (modified Rankin Scale (mRS) 0–2) at 90 days than SR (OR = 1.4, 95%CI 1.1–1.9), whereas SRA did not (SUCRA 90.5, 17.4, 42.1%).

**Conclusion:**

For acute PCS, a first-line CA strategy yielded better results in terms of final successful reperfusion and 90-day functional independence compared to SR. As the combined technique, first-line SRA was associated with superior first-pass and final reperfusion outcomes compared to SR. However, no significant difference was observed in functional independence achieved by first-line SRA compared to the other two strategies. Further high-quality studies are warranted.

## Introduction

Posterior circulation strokes (PCS), particularly basilar artery occlusion (BAO), are associated with unfavorable clinical outcomes (1, 2). Although two randomized controlled trials (RCTs), the BEST (3) and BASICS (4) trials, did not show significant differences between endovascular therapy and medical therapy for acute PCS, accumulating evidence supported the potential essential role of endovascular thrombectomy. Both BAOCHE (5) and ATTENTION (6) trials, as well as a meta-analysis of the above four RCTs (7, 8), have suggested that endovascular treatment might improve clinical prognosis compared to best medical care. Consequently, endovascular therapy is widely used for acute PCS in clinical practice.

As first-line strategies, all the following three thrombectomy techniques are commonly applied: contact aspiration (CA), stent retriever (SR), as well as a combined technique of SR and CA (SRA). The SR technique was primarily employed in the BEST (3) and BAOCHE (5) trials, while CA was widely used in the BASICS (4) and ATTENTION (6) trials. Previous meta-analyzes have indicated that CA might be superior to SR in terms of reperfusion and clinical outcomes (9, 10). Furthermore, it is worth mentioning that SRA was used for nearly half of the patients in the ATTENTION trials (6). However, there is still an unclear comparison between these three first-line strategies, especially regarding the efficacy and safety of SRA. Therefore, this study was aimed to perform a network meta-analysis.

## Methods

The systematic review was reported in accordance with the PRISMA (Preferred Reporting Items for Systematic Reviews and Meta-Analyzes) guidelines (11).

### Literature inclusion and risk of bias assessment

Studies reporting reperfusion and clinical outcomes of different first-line strategies for acute PCS were included. A systematic search was conducted in three databases (PubMed, Embase, and Cochrane) for literature published before April 26, 2023. The PubMed search algorithm used was as follows: (((posterior circulation stroke*[Title/Abstract]) OR (basilar artery occlusion [Title/Abstract])) AND (stent*[Title/Abstract])) AND (aspiration*[Title/Abstract]). Exclusion criteria were as follows: (1) secondary review papers; (2) conference abstracts; (3) studies without a comparison between groups; (4) sample size in either group less than 10 cases.

Risk of bias was assessed using the Newcastle Ottawa scale for cohort studies (12). Factors indicating a low risk of bias included well-defined selection criteria, comparable baseline stroke severity, and independent assessment of recanalization and clinical outcomes. Two investigators independently conducted the literature search, selection, and risk of bias assessment. Discrepancies were resolved through discussion and consensus.

### Data extraction

The following clinical characteristics were extracted: (1) baseline characteristics: patient number, age, sex, occlusion site, stroke etiology, duration from onset to puncture, National Institutes of Health Stroke Scale (NIHSS) score on admission, intravenous thrombolysis; (2) recanalization outcomes: final successful recanalization [modified Thrombolysis In Cerebral Infarction (mTICI) 2b-3], final excellent reperfusion (mTICI 2c-3), final complete reperfusion (mTICI 3), complete reperfusion after first-pass of maneuvers (first pass effect, FPE), successful reperfusion after first-pass of maneuvers (mFPE), number of passes, new-territory embolic event, rescue therapy, duration from puncture to reperfusion, procedure duration; (3) clinical outcomes: functional independence [modified Rankin Scale (mRS) 0–2] and mortality at 90 days, symptomatic intracranial hemorrhagic event (sICH).

### Outcome variables and statistical analysis

The following primary outcomes were compared: final successful reperfusion, FPE, and 90-day functional independence. Secondary outcomes included final excellent reperfusion, final complete reperfusion, mFPE, sICH, and mortality. Random-effect network meta-analyzes were performed using Bayesian Markov chain Monte Carlo modeling. Forest plots were utilized to present pooled estimates, and odds ratios (OR) with 95% confidence intervals (CI) were calculated. Rankograms were constructed, and the surface under the cumulative ranking curve (SUCRA) was calculated to determine treatment ranking probabilities. Heterogeneity between studies was assessed using the I^2^ statistic, and I^2^ > 50% indicated moderate to high heterogeneity. Statistical analyzes were conducted using R software (V 3.6.2).

## Results

### Literature search inclusion and overview

**Figure 1:**
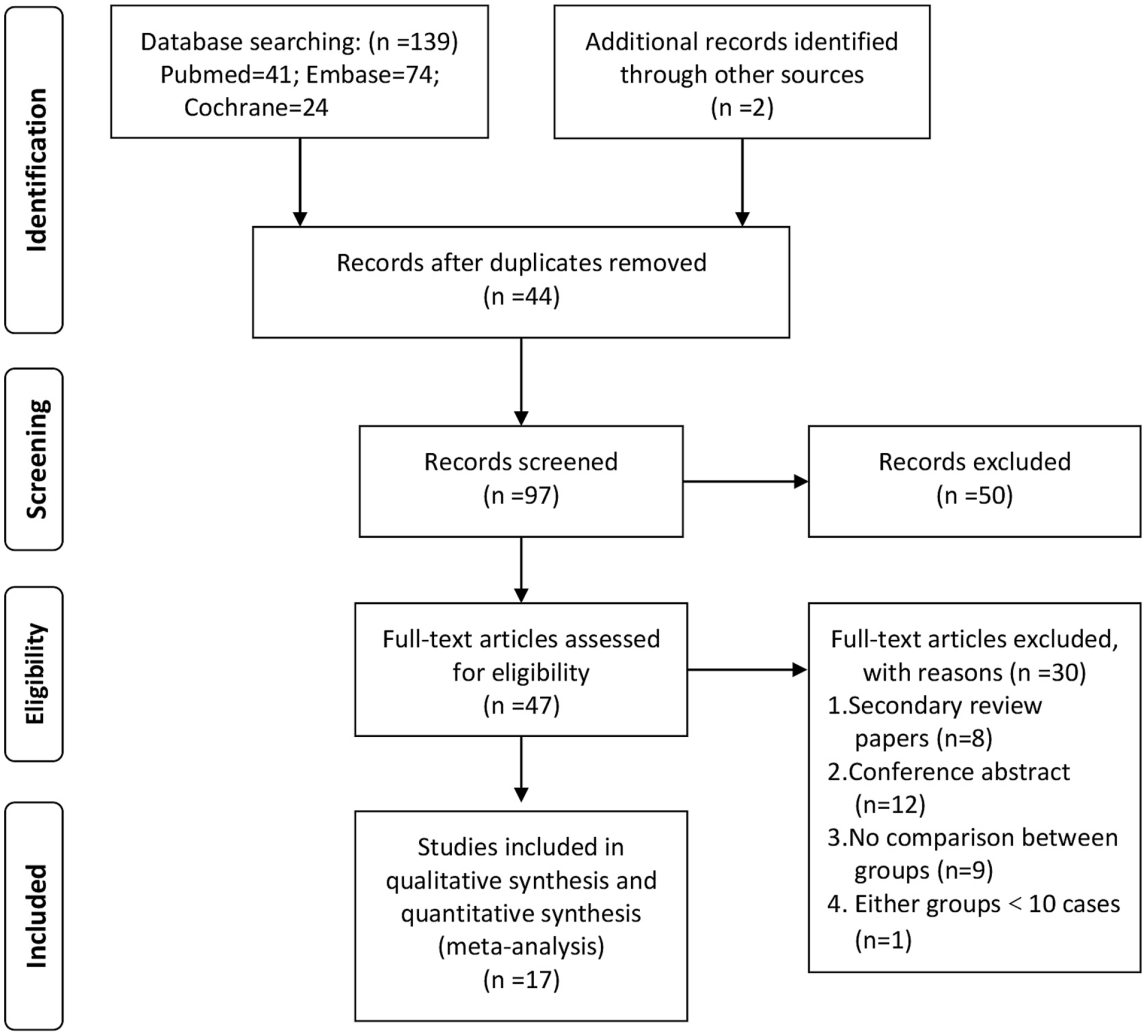
PRISMA search flow diagram.

**Figure 2:**
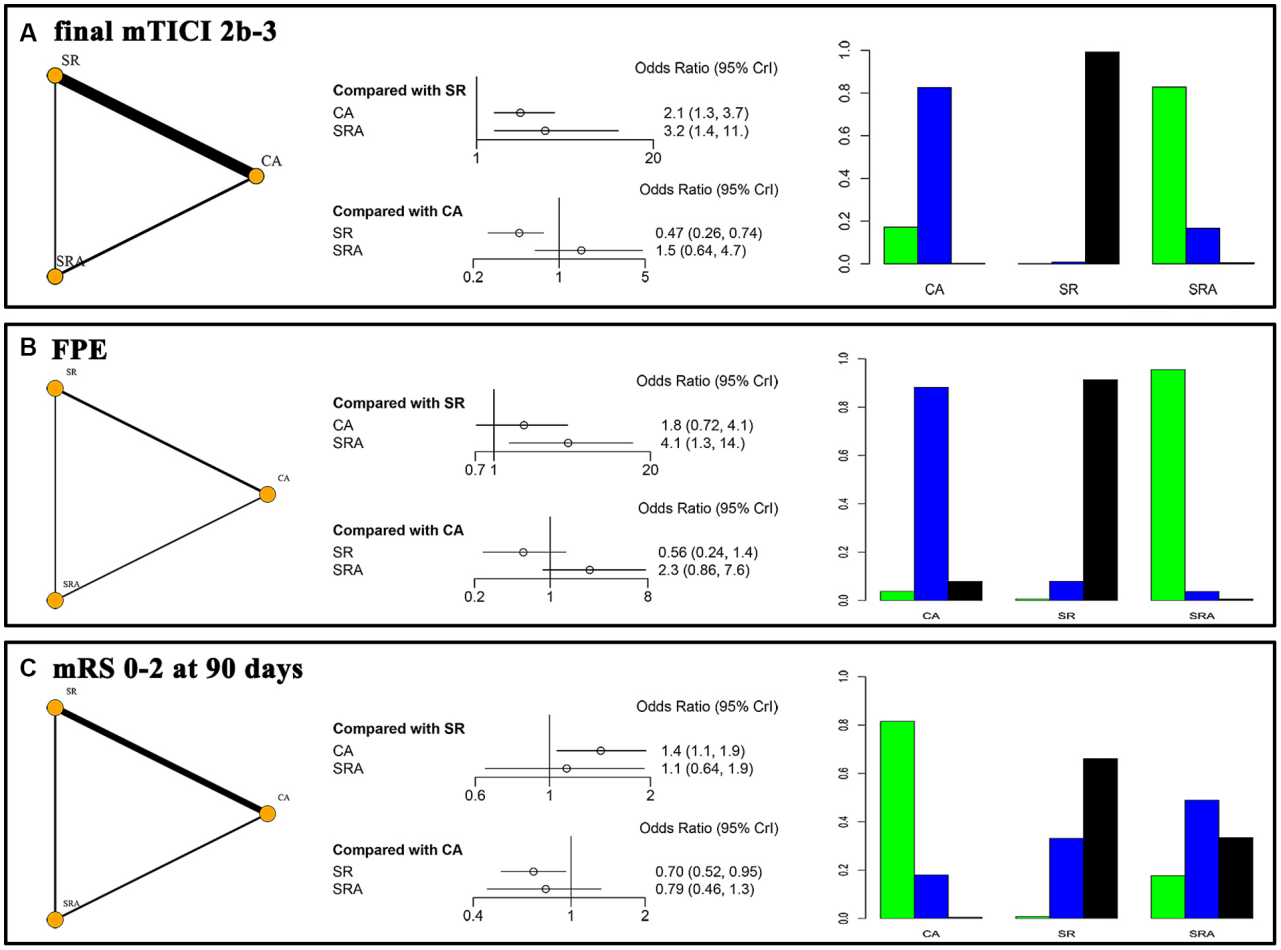
Network plots, forest plots, and rankograms for the primary outcomes. **(A)** final mTICI 2b/3; **(B)** FPE, first pass complete reperfusion; **(C)** mRS 0–2 at 90 days.

**Figure 3:**
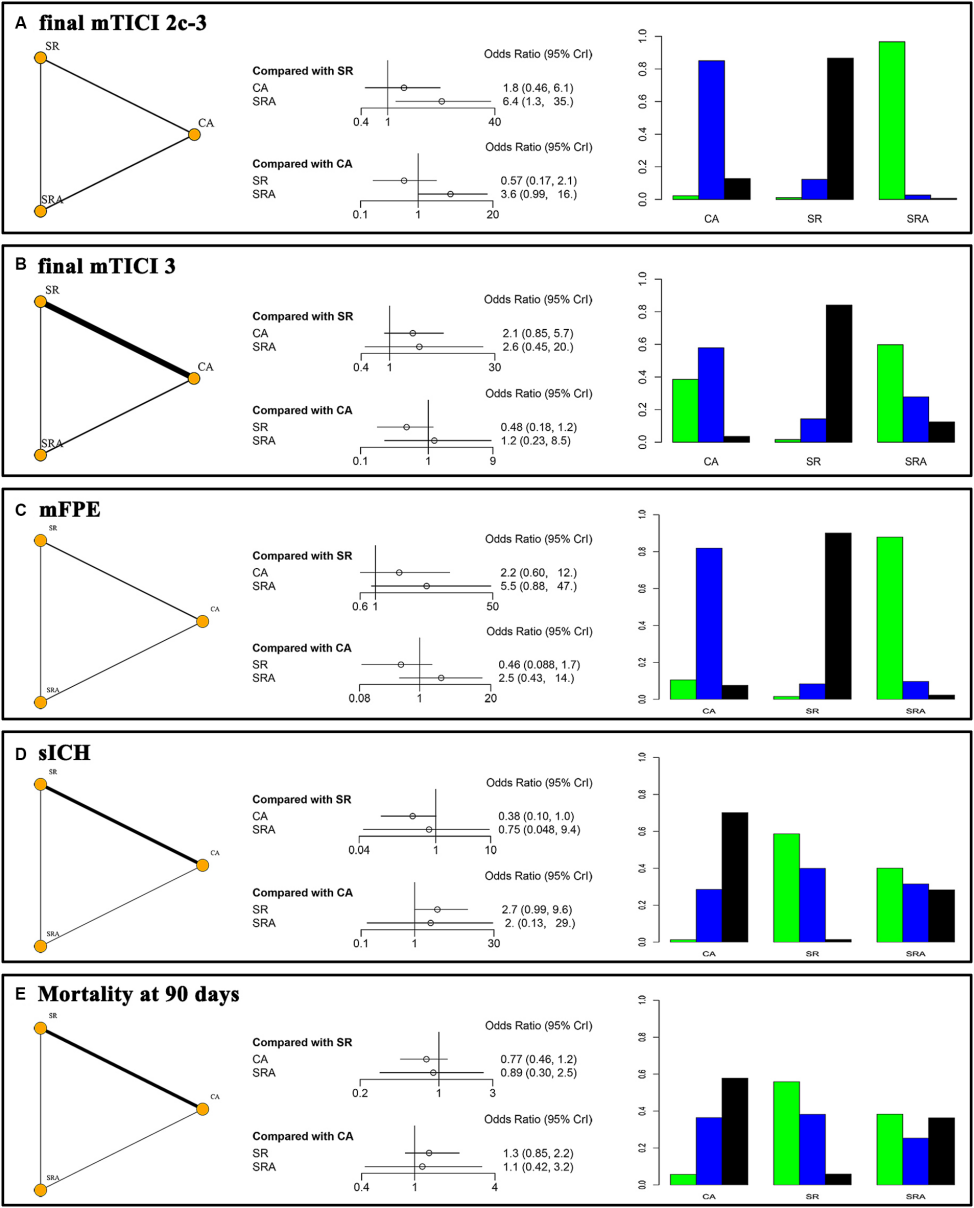
Network plots, forest plots, and rankograms for the secondary outcomes. **(A)** final mTICI 2c/3; **(B)** final mTICI 3; **(C)** mFPE, first pass successful reperfusion; **(D)** sICH, symptomatic intracranial hemorrhage; **(E)** mortality at 90 days.

Figure 1 presents the flow diagram for the literature search and selection process. Seventeen observational studies were included in this analysis (13–29). The first-line strategies of CA, SR and SRA were used in 645, 850, and 166 patients, respectively. Table 1 provides an overview of the clinical characteristics of the included studies. Network plots, forest plots, and rankograms are displayed in Figures 2, 3. The pooled estimates and ranking probabilities are summarized in Table 2. The risk of bias assessment and the results of the heterogeneity test are presented in the Supplementary material.

**Table 1:** Clinical characteristics of included studies.

Study	Country and centers	Risk of Bias (NOS)	Total number	Occlusion site (%)	First-line strategy	No. of patients	Stroke etiology (%)	Age (mean or median, y)	Male (%)	Onset to puncture time (median, min)	NIHSS (median)	IV-tPA (%)	Recanalization time (median, min)	Procedure duration (median, min)	Rescue therapy (%)	mTICI 2b-3 (%)	mTICI 2c-3 (%)	mTICI 3 (%)	mFPE (%)	FPE (%)	No. of passess (median)	New-territory embolic event (%)	sICH (%)	mRS 0–2 at 90-d (%)	Motality at 90-d (%)
Son et al. (13)	**Korea** **1 center**	**6**	**31**	**BA**	**CA**	**18**	**CE (69.2)**	**66.4**	**77.8**	127.2*	**21.3**	**50.0**	**-**	62.3*	**-**	**100.0**	**-**	72.2*	**-**	**-**	**-**	**-**	**0**	**44.4**	**38.9**
					**SR**	**13**	**CE (55.6)**	**69.8**	**53.8**	231.2*	**27.3**	**38.5**	**-**	101.9*	**-**	**84.6**	**-**	23.1*	**-**	**-**	**-**	**-**	**0**	**38.5**	**46.2**
Mokin et al. (14)	**United States** **Multi-centers**	**5**	**100**	**Distal BA (46.0)** **Other BA (48.0)** **VA (21.0)** **PCA (31.0)**	**CA**	**42**	**AF (28.0)**	**63.5**	**67.0**	**562**	**19.2**	**32.0**		**46**	**14.3**	**83.3**	**-**	**-**	**-**	**-**	**-**	**-**	**5.0**	**33.0**	**-**
					**SR**	**58**								**56**	**22.4**	**77.6**	**-**	**-**	**-**	**-**	**-**	**-**		**36.2**	**-**
Gerber et al. (15)	**Germany** **1 center**	**6**	**33**	**VA (6.1)** **Proximal BA (15.2)** **Middle BA (60.6)** **Distal BA (18.2)**	**CA**	**20**	**AF (30.0)**	**62.8**	**70.0**	**321**	**18**	**60.0**		55*	**-**	**85.0**	**-**	75.0*	**-**	**-**	**-**	**10.0**	**-**	45.0*	**20.0**
					**SR**	**13**	**AF (15.4)**	**63.2**	**61.5**	**353**	**25**	**84.6**		97*	**-**	**69.2**	**-**	46.2*	**-**	**-**	**-**	**23.1**	**-**	7.7*	**30.8**
Gory et al. (16)	**France** **3 centers**	**8**	**100**	**Proximal BA (24.5)** **Middle BA (22.6)** **Distal BA (52.8)**	**CA**	**46**	**CE (37.2), LAA (32.6)**	**61**	**58.7**	**342**	**14**	**50.0**	45*		26.1*	**87.0**	**-**	**54.3**	**-**	**-**	**2**	2.2*	**0**	**40.0**	**46.7**
					**SR**	**54**	**CE (27.1), LAA (33.3)**	**67**	**63.0**	**276**	**20**	**40.7**	56*		3.7*	**72.2**	**-**	**31.5**	**-**	**-**	**2**	18.5*	**4.0**	**34.0**	**42.0**
Kang et al. (17)	**Korea** **3 centers**	**7**	**212**	**BA**	**CA**	**67**	**CE (47.6) LAA (38.7)** **ICAS (25.9)**	**71**	**56.6**	**242**	20*	**30.7**	**-**	**44**	**22.4**	**94.0**	**-**	**61.2**	**-**	**-**	**-**	**-**	**-**	**40.3**	**16.4**
					**SR**	**145**					16*		**-**	**38**	**22.1**	**90.3**	**-**	**64.1**	**-**	**-**	**-**	**-**	**-**	**46.9**	**15.9**
Maus et al. (18)	**Germany** **1 center**	**5**	**39**	**BA, PCA**	**CA**	**21**	**-**	**-**	**-**	**-**	**-**	**-**	**39**	**-**	**-**	**61.9**	**33.3#**	**14.3#**	**33.3#**	**9.5#**	**2#**	**-**	**-**	**-**	**-**
					**SRA**	**18**	**-**	**-**	**-**	**-**	**-**	**-**	**46**	**-**	**-**	**88.9**	**77.8#**	**66.7#**	**66.7#**	**55.6#**	**1#**	**-**	**-**	**-**	**-**
Alawieh et al. (19)	**United States and German** **STAR Collaboration**	**8**	**296**	**BA (80.8)** **P2/3 (9.6)**	**CA**	**127**	**AF (29.1)**	**66**	**51.2**	**500**	**17**	**38.**	**-**	40*#	**-**	**87.2**	**-**	**55.2#**	**-**	**-**	**2**	**-**	**-**	38.8*#	31.9*#
					**SR**	**107**	**AF (22.5)**	**64**	**56.1**	**550**	**18**	**39.4**	**-**	76*	**-**	**78.5**	**-**	**48.6**	**-**	**-**	**2**	**-**	**-**	20.5*	45.8*
					**SRA**	**62**	**AF (32.8)**	**70**	**46.8**	**380**	**15**	**38.3**	**-**	**57#**	**-**	**80.4**	**-**	**35.7#**	**-**	**-**	**2**	**-**	**-**	**28.0#**	**48.0#**
Choi et al. (20)	**Korea** **1 center**	**6**	**50**	Distal BA (66.8)*	**CA**	**16**	**AF (43.7)**	**65**	**62.5**	**125**	**19.5**	**31.2**	**-**	28*	**-**	**87.5**	**-**	**-**	**-**	68.8*	1*	**-**	**6.3**	**56.3**	**0**
				Distal BA (38.2)*	**SR**	**34**	**AF (20.5)**	**69**	**47.0**	**140**	**21.5**	**50.0**	**-**	65*	**-**	**73.5**	**-**	**-**	**-**	38.2*	2*	**-**	**8.8**	**35.3**	**17.6**
Baik et al. (21)	**Korea** **2 centers**	**8**	**161**	**Proximal BA (24.8)** **Middle BA (26.1)** **Distal BA (49.1)**	**CA**	**43**	**AF (39.5)**	**72**	**62.8**	**298**	**21**	**25.6**	**-**	33*	**30.2**	**86.0**	**-**	69.8*	**-**	39.5*	1*	**-**	**0**	**39.5**	**9.3**
					**SR**	**118**	**AF (37.3)**	**73**	**56.8**	**277**	**16**	**25.4**	**-**	56*	**28.0**	**82.2**	**-**	47.5*	**-**	19.5*	3*	**-**	**11.0**	**32.2**	**22.2**
Kaneko et al. (22)	**Japan** **12 centers**	**5**	**73**	**Proximal BA (23.3)** **Middle BA (37.0)** **Distal BA (37.0)**	**CA**	**21**	**CE (61.6), LAA (23.3)**	**-**	**-**	**-**	**-**	**-**	**-**	**-**	**-**	**-**	**-**	**-**	**-**	**-**	**-**	**-**	**-**	**38.1**	**-**
					**SR**	**38**		**-**	**-**	**-**	**-**	**-**	**-**	**-**	**-**	**-**	**-**	**-**	**-**	**-**	**-**	**-**	**-**	**39.5**	**-**
					**SRA**	**10**		**-**	**-**	**-**	**-**	**-**	**-**	**-**	**-**	**-**	**-**	**-**	**-**	**-**	**-**	**-**	**-**	**10.0**	**-**
Monteiro et al. (23)	**United States** **2 centers**	**5**	**83**	**Proximal BA (25.3)** **Middle BA (31.3)** **Distal BA (43.4)**	**CA**	**23**	**AF (14.5), ICAS (28.9)**	**-**	**-**	**-**	**-**	**-**	**-**	**-**	**-**	**-**	**-**	**-**	**36.8**	**34.8**	**-**	**-**	**-**	**39.1**	**-**
					**SR**	**20**		**-**	**-**	**-**	**-**	**-**	**-**	**-**	**-**	**-**	**-**	**-**	**30.0**	**35.0**	**-**	**-**	**-**	**30.0**	**-**
					**SRA**	**40**		**-**	**-**	**-**	**-**	**-**	**-**	**-**	**-**	**-**	**-**	**-**	**55.0**	**45.0**	**-**	**-**	**-**	**32.5**	**-**
Onodera et al. (24)	**Japan** **1 center**	**7**	**34**	**BA (88.2), VA (11.8)**	**CA**	**23**	**AF (65.2)**	**74.8**	**73.9**		**22.7**	**34.8**	40*	**-**	**13.0**	**95.7**		**73.9**	**-**	**56.5**	**1**	**-**	**8.7**	**52.2**	
					**SR**	**11**	**AF (36.4)**	**72.1**	**63.6**		**21**	**36.4**	65*	**-**	**27.3**	**90.9**		**36.4**	**-**	**36.4**	**2**	**-**	**9.1**	**27.3**	
Yuan et al. (25)	**China** **1 center**	**6**	**82**	**BA (75.6), VA (24.4)**	**CA**	**37**	**CE (67.6), LAA (29.7)**	**63**	**59.5**	**300**	**21**	**18.9**	45*	**-**	32.4*	**86.5**	**-**	**-**	**-**	**-**	**-**	**2.7**	**8.1**	**37.8**	**32.4**
					**SR**	**45**	**CE (44.4), LAA (31.1)**	**66**	**53.3**	**295**	**22**	**15.6**	63*	**-**	8.9*	**84.4**	**-**	**-**	**-**	**-**	**-**	**13.3**	**13.3**	**35.6**	**35.6**
Bernsen et al. (26)	**Netherlands** **MR CLEAN**	**8**	**205**	**VA (3.9)** **BA (40.0)** **BA + PCA (38.5)** **PCA (13.2)**	**CA**	**71**	**CE (19.7), LAA (33.8)**	**60**	**57.8**	**230**	**19**	**42.2**		49*	**-**	87*	62*	**50**	**62**	**-**	**-**	**-**	**3**	44*	**46**
					**SR**	**134**	**CE (30.6), LAA (29.1)**	**66**	**55.2**	**262**	**20**	**50.0**		69*	**-**	73*	48*	**37**	**49**	**-**	**-**	**-**	**4**	29*	**42**
Lin et al. (27)	**China** **2 centers**	**5**	**38**	**Distal BA**	**CA**	**17**	**AF (58.8)**	**67**	**58.8**	**-**	16*	**5.9**	20*	**-**	5.9*	**100**	**-**	**-**	**94.1**	**-**	**-**	**0**	**0**	**-**	**-**
					**SR**	**21**	**AF (68.2)**	**65**	**68.2**	**-**	21*	**22.7**	41*	**-**	40.9*	**63.6**	**-**	**-**	**54.5**	**-**	**-**	**22.7**	**9.1**	**-**	**-**
Abdelrady et al. (28)	**France** **2 centers**	**7**	**128**	**VA (11)** **Proximal BA (22)** **Middle BA (23)** **Distal BA (44)**	**CA**	**53**	**AF (21.1), ICAS (24.2)**	**-**	**-**	**351**	**15**	**35.9**	**-**	**-**	**19.5**	**85.2**	**64.8**	**-**	**-**	**28.3**	**-**	**-**	**3.8**	**-**	**-**
					**SR**	**39**												**-**	**-**	**25.6**	**-**	**-**	**5.1**	**-**	**-**
					**SRA**	**36**												**-**	**-**	**50.0**	**-**	**-**	**5.6**	**-**	**-**
Abdelrady et al. (29)	**France** **2 centers**	**6**	**116**	**VA/Proximal BA (38)** **Middle BA (14)** **Distal BA (48)**	**CA**	**50**	**CE (34.5), LAA (41.4)**	**68**	**64.7**	**376**	**15**	**33.6**	**-**	**50**	**20.0**	**84.0**	**62.0**	**-**	**-**	**-**	**-**	**-**	**-**	**-**	**-**
					**SR**	**33**									**15.2**	**69.7**	**45.5**	**-**	**-**	**-**	**-**	**-**	**-**	**-**	**-**
					**SRA**	**33**									**27.3**	**97.0**	**78.8**	**-**	**-**	**-**	**-**	**-**	**-**	**-**	**-**

The data of age, onset to puncture time, procedure duration, NIHSS on admission, and No. of passes are shown as mean value or median. ^*^Significant difference between first-line CA and SR (*p* < 0.05). # significant difference between first-line SRA and CA (*p* < 0.05). NOS, Newcastle Ottawa scale; BAO, basilar artery; VA, vertebral artery; PCA, posterior cerebral artery; SRA, combined stent retriever and contact aspiration; CA, contact aspiration; SR, stent retriever; P2/3, second or third segment of the posterior cerebral artery; CE, cardioembolism; LAA, large artery atherosclerosis; AF, atrial fibrillation; ICAS, intracranial atherosclerotic stenosis; NIHSS, National Institutes of Health Stroke Scale; IV, intravenous; mTICI, modified Thrombolysis in Cerebral Infarction; mFPE, sICH, symptomatic intracranial hemorrhage; mRS, modified Rankin Scale. Mokin et al. showed the occlusion site involved, so the total proportion exceeds 100%. Gerber et al. used arterial occlusive lesion (AOL) score to assess recanalization outcomes; AOL 2–3 and AOL 3 were defined as successful and complete recanalization, respectively. Besides, they reported the proportion of mRS 0–3 and mortality during hospitalization without the 90-day follow-up.

**Table 2:** Summary of the network meta-Analysis.

Outcomes	First-line strategy	OR (95%CI)	*I* ^2^	OR (95%CI)	*I* ^2^	SURCA (rank)
Successful reperfusion (final mTICI 2b/3)	SRA	3.2 (1.4–11.0)	74.8%	1.5 (0.64–4.7)	63.8%	91.1% (1)
	CA	2.1 (1.3–3.7)	0%	Reference	-	58.5% (2)
	SR	Reference	-	-	-	0.4% (3)
FPE	SRA	4.1 (1.3–14.0)	91.3%	2.3 (0.86–7.6)	53.8%	97.4% (1)
	CA	1.8 (0.72–4.1)	0%	Reference	-	48.0% (2)
	SR	Reference	-	-	-	4.6% (3)
Functional independence (mRS 0–2) at 90 days	SRA	1.1 (0.64–1.9)	0%	0.79 (0.46–1.3)	0%	42.1% (2)
	CA	1.4 (1.1–1.9)	0%	Reference	-	90.5% (1)
	SR	Reference	-	-	-	17.4% (3)
Excellent reperfusion (final mTICI 2c/3)	SRA	6.4 (1.3–35.0)	0%	3.6 (0.99–16.0)	30.5%	98.0% (1)
	CA	1.8 (0.46–6.1)	0%	Reference	-	44.7% (2)
	SR	Reference	-	-	-	7.2% (3)
Complete reperfusion (final mTICI 3)	SRA	2.6 (0.45–20.0)	92.2%	1.2 (0.23–8.5)	92.9%	73.7% (1)
	CA	2.1 (0.85–5.7)	58.0%	Reference	-	67.5% (2)
	SR	Reference	-	-	-	8.8% (3)
mFPE	SRA	5.5 (0.88–47.0)	0%	2.5 (0.43–14.0)	0%	92.8% (1)
	CA	2.2 (0.60–12.0)	51.3%	Reference	-	51.4% (2)
	SR	Reference	-	-	-	5.7% (3)
sICH	SRA	0.75 (0.05–9.4)	Na	2.0 (0.13–29.0)	Na	55.9% (2)
	CA	0.38 (0.1–1.0)	0%	Reference	-	15.6% (3)
	SR	Reference	-	-	-	78.6% (1)
Mortality	SRA	0.89 (0.30–2.5)	Na	1.1 (0.42–3.2)	Na	51.0% (2)
	CA	0.77 (0.46–1.2)	14.2%	Reference	-	24.0% (3)
	SR	Reference	-	-	-	75.0% (1)

mTICI, modified Thrombolysis in Cerebral Infarction; FPE, first pass complete reperfusion; mRS, modified Rankin Scale; mFPE, first pass successful reperfusion; sICH, symptomatic intracranial hemorrhage; SRA, combined stent retriever and contact aspiration; CA, contact aspiration; SR, stent retriever; OR, odds ratio; CI, confidence interval.

### Primary outcomes

#### Final successful reperfusion and FPE

Both first-line SRA (OR = 3.2, 95% CI 1.4–11.0) and CA (OR = 2.1, 95%CI 1.3–3.7) demonstrated superiority over SR in achieving final mTICI 2b/3 reperfusion. There was no significant difference between first-line SRA and CA (OR = 1.5, 95%CI 0.64–4.7). The SUCRA values for SRA, CA, and SR were 91.1, 58.5, and 0.4%, respectively (Figure 2A). Compared with SR, first-line SRA also showed an advantage in achieving FPE (OR = 4.1, 95%CI 1.3–14.0), while CA did not (OR = 1.8, 95%CI 0.72–4.1). The SUCRA values for SRA, CA, and SR were 97.4, 48.0, and 4.6%, respectively (Figure 2B).

#### 90-day functional independence

Regarding clinical prognosis, the first-line CA strategy was associated with greater functional independence at 90 days compared to SR (OR = 1.4, 95%CI 1.1–1.9). However, there was no significant difference between the SRA technique and either CA (OR = 0.79, 95%CI 0.46–1.3) or SR (OR = 1.1, 95%CI 0.64–1.9). The SUCRA values for SRA, CA, and SR were 42.1, 90.5, and 17.4%, respectively (Figure 2C).

### Secondary outcomes

#### Final excellent, complete reperfusion and mFPE

The first-line SRA strategy demonstrated significantly superior outcomes in achieving final mTICI 2c/3 reperfusion compared to SR (OR = 6.4, 95%CI 1.3–35.0), and it had a better tendency than CA (OR = 3.6, 95%CI 0.99–16.0). The SUCRA values for SRA, CA, and SR were 98.0, 44.7, and 7.2%, respectively (Figure 3A). There were no significant differences among the three first-line strategies regarding achieving final complete reperfusion, with SUCRA values of 73.7, 67.5, and 8.8%, respectively (Figure 3B). Similarly, there were no significant differences in mFPE outcomes among these three techniques, with SUCRA values of 92.8, 51.4, and 5.7%, respectively (Figure 3C).

#### sICH

The first-line CA strategy was associated with a lower incidence of sICH compared to SR (OR = 0.38, 95%CI 0.1–1.0). However, the SRA technique did not show a significant difference compared to either CA (OR = 2.0, 95%CI 0.13–29.0) or SR (OR = 0.75, 95%CI 0.05–9.4). The SUCRA values for SRA, CA, and SR were 55.9, 15.6, and 78.6%, respectively (Figure 3D).

#### Mortality

There were no significant differences in mortality at 90 days among the first-line SRA, CA, and SR strategies, with SUCRA values of 51.0, 24.0, and 75.0%, respectively (Figure 3E).

## Discussion

This network meta-analysis included 17 observational studies comprising 1,661 cases of acute PCS. The pooled results indicated that the first-line SRA or CA strategies achieved higher rates of final successful reperfusion compared to the SR technique (SUCRA values of 91.1, 58.5, and 0.4%). Additionally, the first-line SRA approach demonstrated advantages in achieving FPE and final excellent reperfusion outcomes compared to the SR technique, while CA did not show such benefits. In terms of the incidence of sICH, the first-line CA strategy had a lower rate compared to SR, whereas no significant difference was observed for SRA (SUCRA values of 15.6, 78.6, and 55.9%). With regard to functional independence at 90 days, the first-line CA strategy appeared to be superior to SR, while SRA did not show a significant difference compared to either CA or SR techniques (SUCRA values of 56.0, 92.0, and 1.9%, respectively).

The present study showed that the first-line CA strategy achieved better final successful reperfusion than SR, which was consistence with the other meta-analysis in for acute PCS (9, 10). However, it was different from the finding in anterior circulation strokes (ACS), for both ASTER (30) and COMPASS trials (31) showing comparable reperfusion outcomes between CA and SR. This discrepancy may be attributed to the straighter characteristics of vertebrobasilar artery. It makes the larger-bore intermediate catheter easier to deliver, and becomes more stable when capture and retriever (32). Besides, SR alone might cause the wiggle of thrombus between the two posterior cerebral arteries, reducing the effectiveness of stentriever. Moreover, CA technique does not require crossing the microguidewire and microcatheter over the thrombus, reducing the risk of vessel perforation and dissection. These factors likely contribute to the efficacy and safety of the CA technique for PCS.

Furthermore, our study favored the first-line SRA strategy in achieving first-pass and final recanalization outcomes compared to SR. On the one hand, the use of an intermediate catheter improved system stability and reduced the difficulty of superselection. On the other hand, the proximal suction of a large-bore catheter could probably improve the reperfusion efficacy of stentriever and reduce the risk of distal embolism. Whereas, this finding was inconsistent with a relevant RCT conducted on ACS, which failed to demonstrate better recanalization outcomes of SRA compared to SR (33). In this RCT study, the use of a balloon-guide catheter was mandatory in SR and SRA groups. Consequently, this difference between PCS and ACS may be attributed to the restricted usage of balloon-guide catheters in PCS cases.

Regarding the comparison between first-line SRA and CA, our study indicated comparable proportions of first-pass and final reperfusion outcomes for PCS. These results differ from the meta-analysis based on ACS (34), which indicated that SRA technique was associated with better recanalization outcomes compared with *CA.* And this kind of difference could be narrowed due to the advantage of CA in PCS discussed. However, it is worth noting that the first-line SRA strategy appeared to be superior to CA in achieving mFPE (SUCRA 92.8 and 51.4%) and FPE (SUCRA 97.4 and 48.0%). In addition to its better recanalization efficacy, this result might partially be due to the higher incidence of intracranial atherosclerotic stenosis (ICAS) in acute PCS cases. The Angel-ACT registry reported that the underlying ICAS accounted for 54.3% (171/315) of acute PCS patients (35), while only 24.8% (282/1139) in acute ACS (36). And this kind of phenomenon was also observed in the Korean population (37). For patients with underlying ICAS, stent-based strategies showed advantages over CA due to the better integration between thrombectomy devices and clots (38).

The positive correlation between recanalization outcomes and clinical prognosis has been reported (16, 28, 39). However, our meta-analysis did not show a significant difference in functional independence between the first-line SRA strategy and the other two strategies. This could be due to the small sample size of the SRA group. Other factors such as age (17) and baseline NIHSS (17, 23, 39) have also shown predictive value for clinical outcomes, but this information was rarely provided in the included studies.

### Limitation

There were several limitations in this study. First, all the included studies were retrospective observational studies with publication and selection bias, potentially leading to unbalanced baseline characteristics among the strategies. Second, the concept of SRA is broad and includes various thrombectomy techniques, such as Solumbra and stent assisted vacuum lock extraction, which may differ in efficacy but were pooled together in our analysis. Third, although the PCS is mainly composed of basilar artery occlusion in this study, the posterior cerebral artery and vertebral artery occlusion were also included, which might increase the heterogeneity. For example, a sub-analysis of the TOPMOST study suggested comparable reperfusion and clinical outcomes between first-line CA and SR for acute P2/3 occlusion (40). Fourth, the safety of the SRA strategy may improve with the use of an intermediate catheter, but this was rarely reported. Only one study provided information on the incidence of symptomatic intracranial hemorrhage, reporting a rate of 5.6% in the SRA group.

## Conclusion

Compared to first-line SR, the CA strategy achieved higher rates of final successful reperfusion and 90-day functional independence, as well as a lower incidence of sICH for patients with acute PCS. The reperfusion outcomes after the first pass were comparable between these two strategies. As a combined approach, the recanalization outcomes after the first pass and at the end of the procedure were significantly better than SR, and also showed advantages over CA from the aspect of SUCRA without significant difference. However, the proportion of functional independence and sICH in the first-line SRA group did not exhibit significant differences compared to the other two strategies. Due to the quality limitations of the included studies, these conclusions should be drawn with caution, and further studies are needed.

## Data availability statement

The raw data supporting the conclusions of this article will be made available by the authors, without undue reservation.

## Author contributions

GY: Conceptualization, Formal analysis, Funding acquisition, Investigation, Methodology, Software, Validation, Writing – original draft. RC: Conceptualization, Data curation, Formal analysis, Investigation, Methodology, Software, Writing – original draft. PC: Data curation, Formal analysis, Investigation, Methodology, Software, Writing – review & editing. HW: Funding acquisition, Validation, Writing – review & editing. DW: Validation, Writing – review & editing. MC: Conceptualization, Funding acquisition, Supervision, Validation, Writing – review & editing. ZL: Conceptualization, Supervision, Validation, Writing – review & editing.
