# Supplementary material for: Network meta-analysis of first-line thrombectomy strategy for acute posterior circulation strokes: a preliminary evaluation for combined approach
Source: Front Neurol. 2023 Nov 3;14:1279233. doi: 10.3389/fneur.2023.1279233 (PMC10654789; doi:10.3389/fneur.2023.1279233)
Supplement: Supplementary file 1 [file Table_1.DOCX]

**SUPPLEMENTARY**

**Table 1. PICOS table**

|  | **PICOS elements** | **Search algorithm** |
| --- | --- | --- |
| **P** | Posterior circulation strokes | (posterior circulation stroke*[Title/Abstract]) OR (basilar artery occlusion [Title/Abstract]) |
| **I/C** | 1. Stent retriever 2. Contact aspiration 3. A combined technique of stent retriever and Contact aspiration | (stent*[Title/Abstract]) AND  (aspiration*[Title/Abstract]) |
| **O** | No restriction | No restriction |
| **S** | No restriction | No restriction |

**Table 2. Risk of bias assessment in each study**

| **Year** | **Author** | **Country** | **Selection** | **Comparability** | **Outcomes** |
| --- | --- | --- | --- | --- | --- |
| 2014 | Son S | Korea | **★★★** | **★** | **★★** |
| 2016 | Mokin M | USA | **★★★** |  | **★****★** |
| 2017 | Gerber JC | Germany | **★★★** | **★** | **★★** |
| 2018 | Gory B | France | **★★★** | **★★** | **★★★** |
| 2018 | Kang DH | Korea | **★★★** | **★** | **★★★** |
| 2019 | Maus V | Germany | **★★★** |  | **★★** |
| 2020 | Alawieh AM | STAR  Collaboration | **★★★** | **★★** | **★★★** |
| 2020 | Choi JW | Korea | **★★★** | **★** | **★★** |
| 2021 | Baik SH | Korea | **★★★** | **★★** | **★★★** |
| 2021 | Kaneko J | Japan | **★★★** |  | **★★** |
| 2021 | Monteiro A | USA | **★★★** |  | **★★** |
| 2021 | Onodera K | Japan | **★★★** | **★** | **★★★** |
| 2021 | Yuan ZZ | China | **★★★** | **★** | **★★** |
| 2022 | Bernsen ML | MR CLEAN | **★★★** | **★★** | **★★★** |
| 2022 | Lin D | China | **★★★** |  | **★★** |
| 2023 | Abdelrady M | France | **★★★** | **★** | **★★★** |
| 2023 | Abdelrady M | France | **★★★** |  | **★★★** |

Note: Risk of bias was assessed by the Newcastle-Ottawa Scale.

**Heterogeneity test**

1. **Final mTICI 2b/3**

**
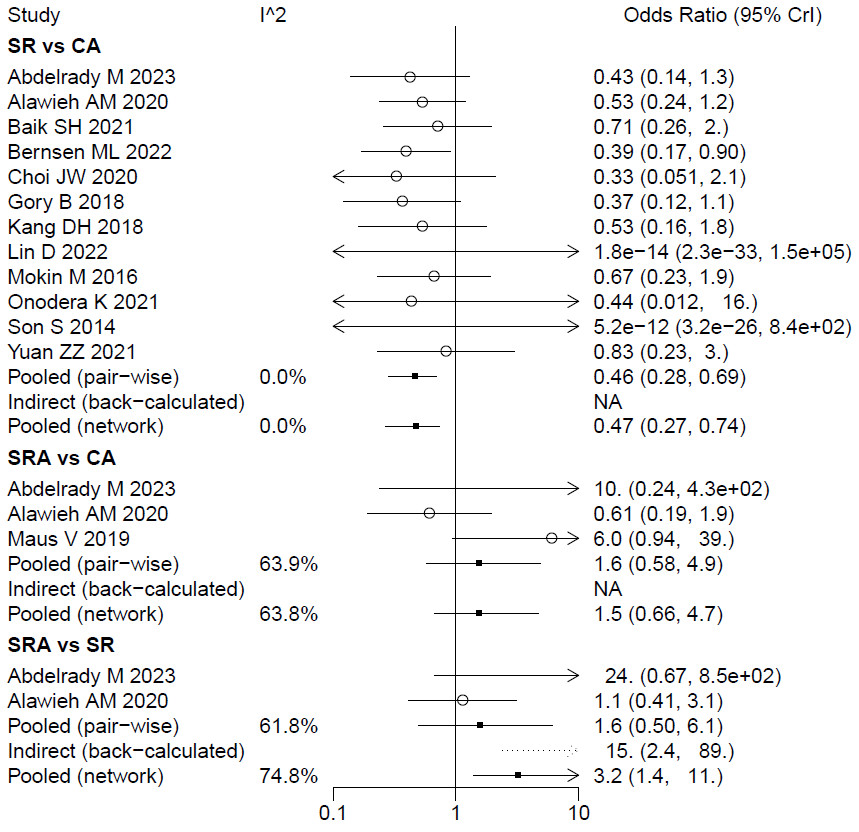
**

1. **FPE, first pass mTICI 3**

**
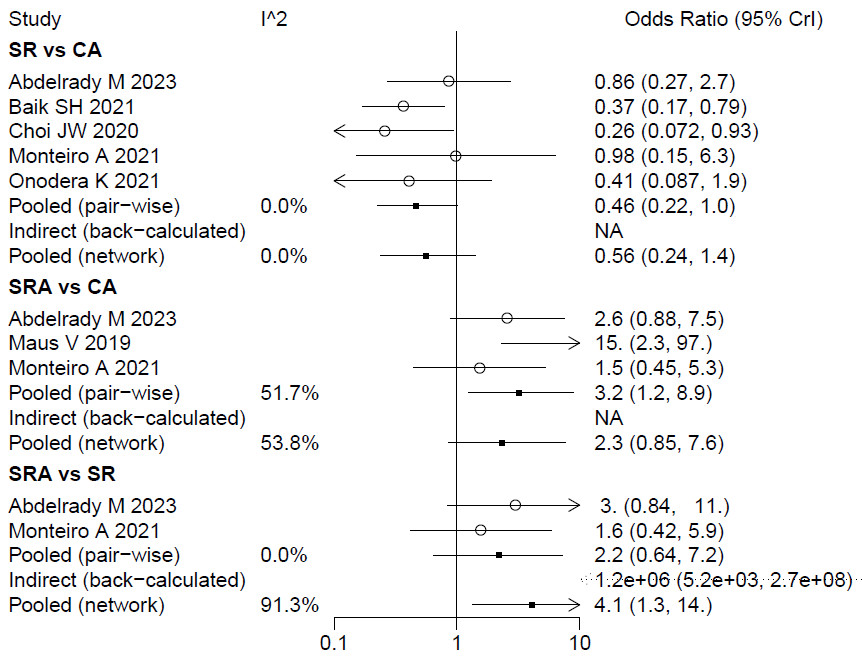
**

1. **mRS 0-2 at 90 days**


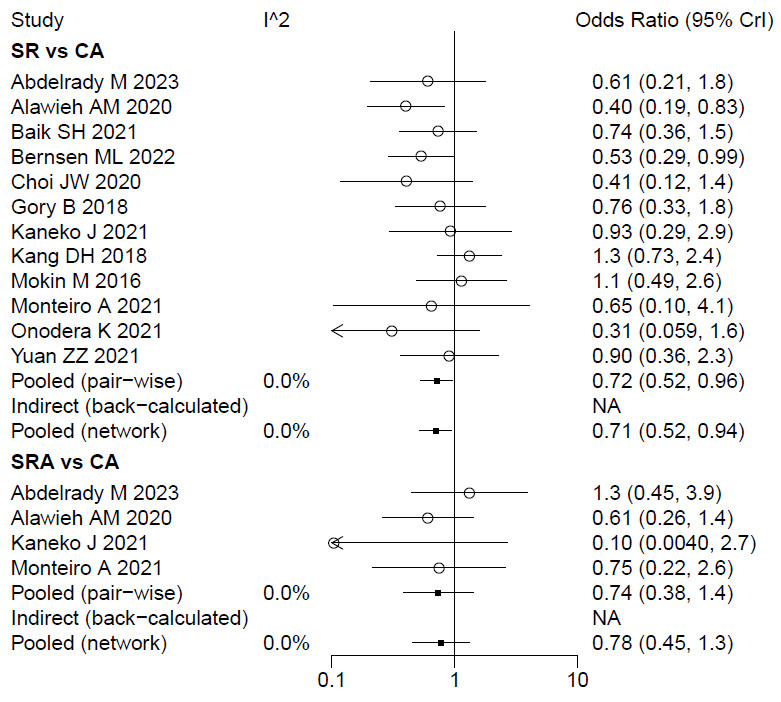


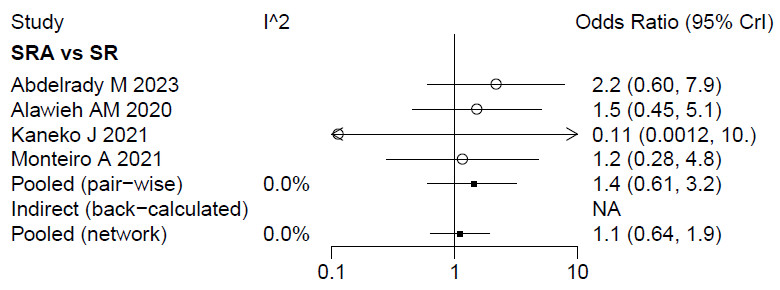


1. **Final mTICI 2c/3**

**
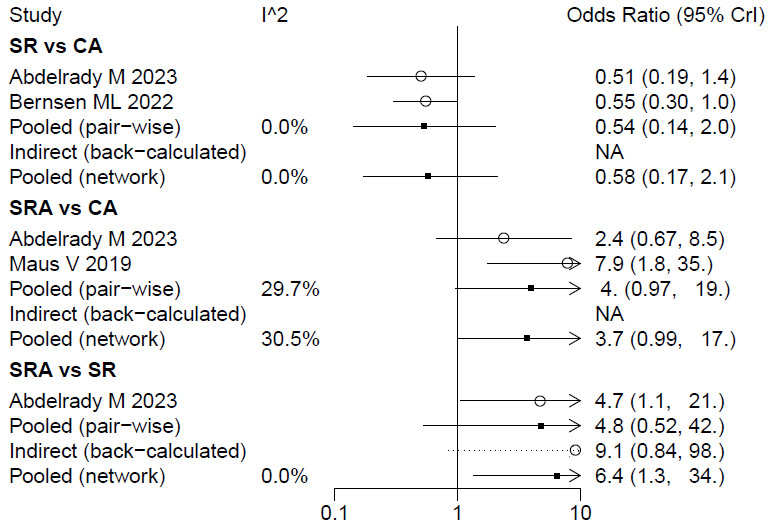
**

1. **Final mTICI 3**

**
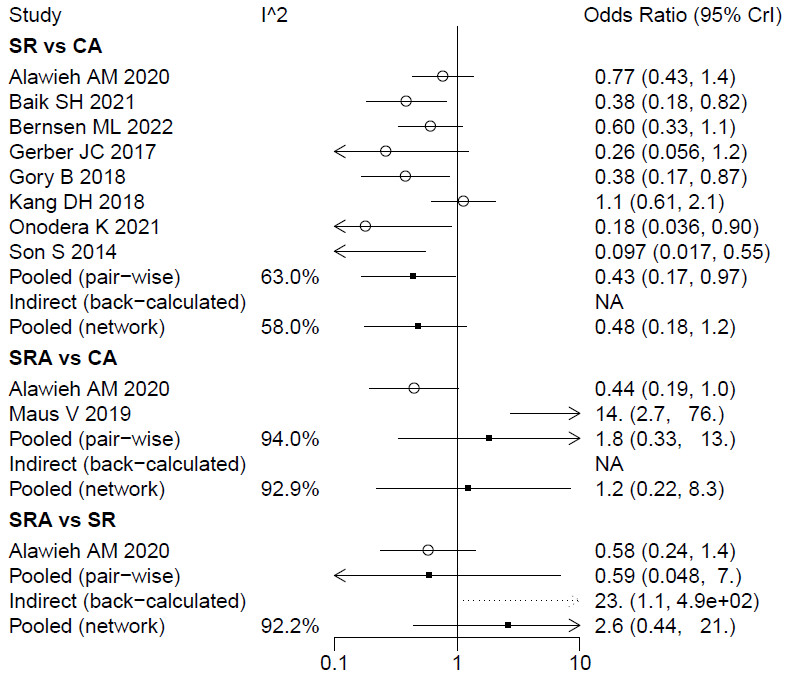
**

1. **mFPE, first pass mTICI 2b/3**


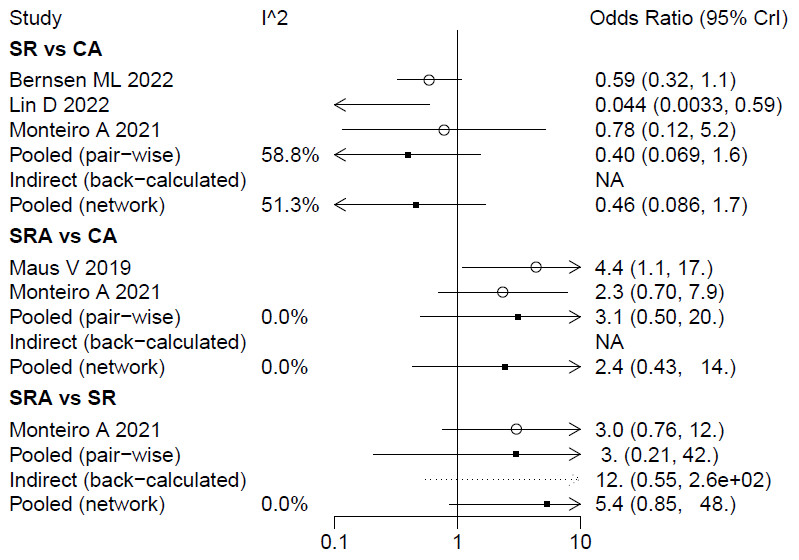


1. **sICH, symptomatic intracranial hemorrhage**


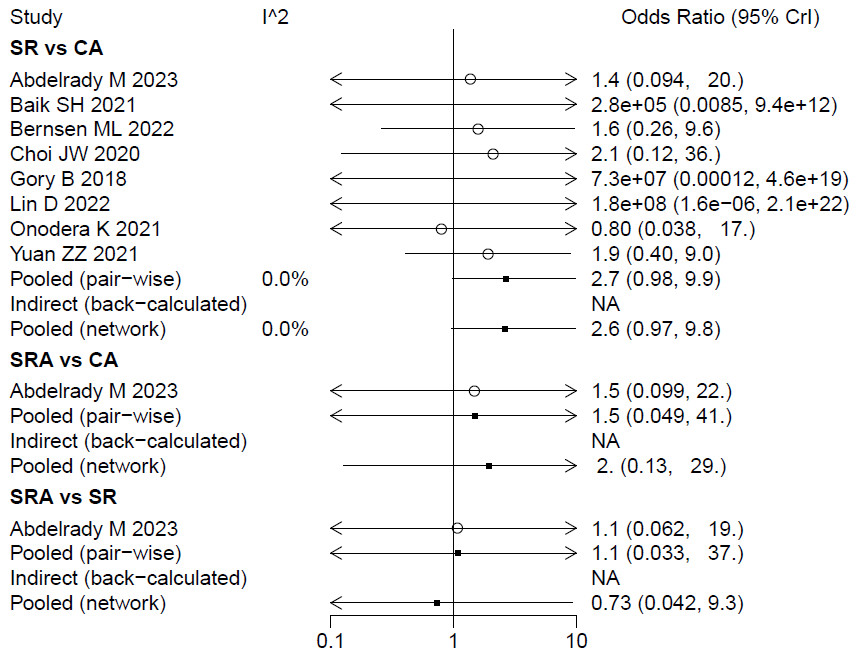


1. **Mortality at 90 days**

**
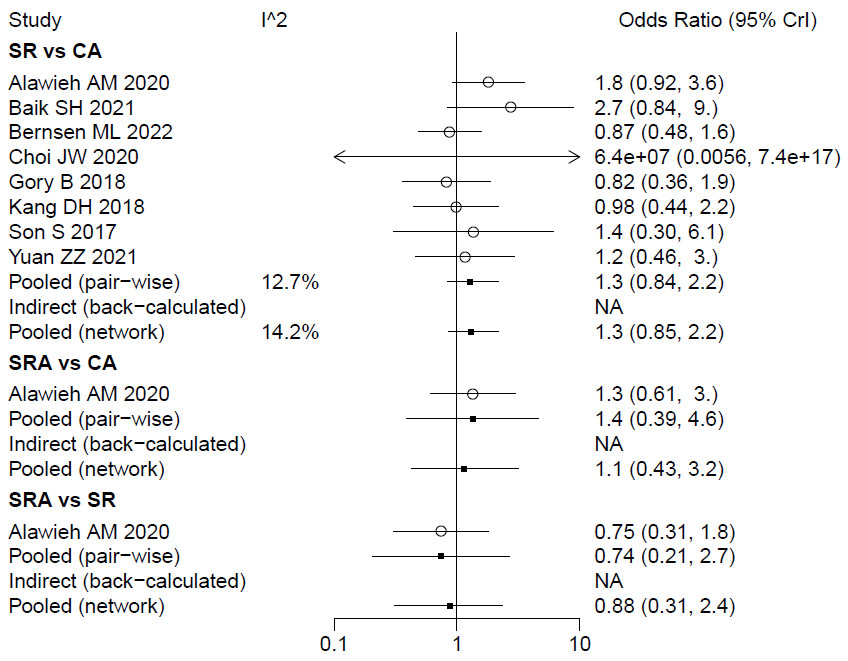
**
